# The relationship between ethmoidal foramina and orbital fat herniation

**DOI:** 10.3906/sag-2102-88

**Published:** 2021-12-09

**Authors:** Fatih ÇANKAL, Mustafa KAYA, İbrahim TEKDEMİR

**Affiliations:** 1 Department of Anatomy, Ankara Medipol University School of Medicine, Ankara, Türkiye; 2 Department of Radiology, Gazi University School of Medicine, Ankara, Türkiye; 3 Department of Anatomy, Ankara University School of Medicine, Ankara, Türkiye

**Keywords:** Anterior ethmoidal foramen, posterior ethmoidal foramen, orbital fat, herniation

## Abstract

**Background/aim:**

The aims of this study are to determine the incidence and more frequent localizations of orbital fat tissue herniation accompanying dehiscences in the medial orbital wall and to investigate the relationship between orbital fat tissue herniations and the anterior and posterior ethmoidal foramina.

**Material and methods:**

One thousand two hundred patients who had undergone computed tomography with a preliminary diagnosis of sinusitis and who had no previous facial, orbital, paranasal sinus surgeries or history of trauma were retrospectively analyzed. The localization of the ethmoidal foramina and orbital fat tissue herniations were marked. In patients with orbital fat tissue herniation, the relationship between the localization of orbital fat tissue herniation and the anterior and posterior ethmoidal foramina was investigated.

**Results:**

The incidence of orbital adipose tissue herniation in our study was 7.9%. Of the 98 herniations on the bilateral medial orbital wall, 60 were in zone 3, and the most common herniation site was zone 3. A statistically significant difference was noted between the localization zone of the anterior ethmoidal foramen and the localization zones of orbital fat tissue herniations (Fisher’s exact test, p < 0.001).

**Conclusion:**

Zone 3 is the weakest area of the medial orbital wall, and zone 3 is the most prone to herniation of fat tissue. The association of orbital fat tissue herniations with the anterior ethmoidal foramen is extremely common. Being cognizant of this finding may help a surgeon better estimate the anatomical view to be met before functional endoscopic sinus surgery as well as to minimize the risk of possible orbital complications, especially anterior ethmoidal artery injury.

## 1. Introduction

Paranasal sinus computed tomography (CT), taken under appropriate conditions, provides detailed information about sinonasal anatomy [1]. One of the rare cases which is encountered while evaluating paranasal sinus tomographies is the herniation of the orbital contents into the ethmoid sinus [2]. This is mostly referred to in the literature as lamina papyracea (LP) dehiscence. It can, however, sometimes be confused with infection or tumoral pathologies by the radiologist on CT images [3]. Magnetic resonance imaging can also be used in herniation cases that are associated with LP dehiscences [4]. Due to its superiority in imaging bone structures, however, CT is required to clearly reveal the bone dehiscence as well as the relationship of the accompanying orbital fat tissue herniation with the ethmoidal foramen. Furthermore, significant steps have been taken in recent years to prevent potential radiation damage with low dose CT imaging protocols using low kilovolt values [5]. The clinical importance of orbital medial wall dehiscences has been reported since orbital structures are vulnerable to injury during functional endoscopic sinus surgery (FESS) [6]. The bone structures that constitute the medial orbital wall are the lateral or orbital, face of the lacrimal bone, the frontal process of the maxillary bone, the LP of the ethmoid bone, and the corpus of the sphenoid bone [7].

Two important structures located in the medial orbital wall are the anterior ethmoidal foramen (AEF) and the posterior ethmoidal foramen (PEF). Damage to the ethmoidal artery and / or veins that pass through the ethmoidal canals during FESS may cause an intraorbital hematoma. Orbital hemorrhage can result in profound visual loss from optic nerve ischemia or retinal ischemia [8]. Also, medial rectus muscle damage, cerebrospinal fluid (CSF) leakage, intracranial infections, and orbital cellulitis may also be triggered by FESS [9, 10, 11].

To the best of our knowledge, there is no study in the literature regarding the contingency of orbital fat tissue herniations accompanying medial orbital wall dehiscences with ethmoidal foramina. In this study, the frequency and localization of fat tissue herniations accompanying dehiscences in the medial orbital wall and the relationship between orbital fat tissue herniations and AEF and PEF are investigated.

## 2. Materials and methods

This study was conducted between February 2018 and February 2020, with a retrospective analysis on a series of 1200 patients (688 males, 512 females) aged between 10 and 70 years old. Scans were made with Siemens brand Somatom Balance VA 10 D model spiral device using 130 kV voltage and 80–120 mAs values, as recommended by the manufacturers. CT images were evaluated with PACS (picture archiving and communication systems). Patients who had previous facial, orbital, and paranasal sinus surgery or trauma, as well as those who had cases of sinusitis thought to cause mild erosion in the ethmoid lamellae, were not included in the study. Scans were made by obtaining contiguous images with a slice thickness of 2.5 mm in the coronal and/or axial plane, and a bone protocol filter was used. Axial sections were planned parallel to the ethmoid roof.

The study protocol was approved by our local institutional ethics committee and all participants provided informed written consents.

**Figure 1:**
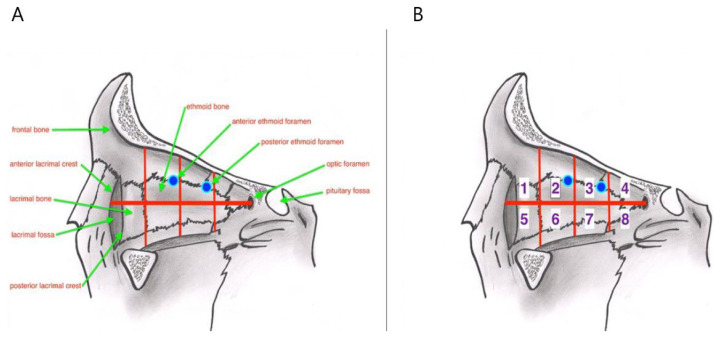
A- Medial orbital wall anatomy. B- Separation of the medial orbital wall in eight coequal zones (Zone 2 blue point: AEF, Zone 3 blue point: PEF).

The medial orbital wall was first divided with a line passing through the middle of the optic foramen and the crista lacrimalis and was labeled either as being upper or lower. This hypothetical line was then divided into four equal parts from front to back. Subsequently, eight zones of the coequal area were obtained (Figures 1A, 1B). The localizations of the ethmoidal foramen and orbital fat tissue herniations were then determined. The dimensions of the orbital fat tissue herniations were measured on all three planes. If an orbital fat tissue herniation occupied more than one zone, the zone where the herniation held the most in size was accepted as being the location of the herniation.

After determining in which zone the herniations accompanying the dehiscence in the medial orbital wall and the anterior and posterior ethmoid foramina were located, the relationship between them was evaluated by Spearman’s rank correlation coefficient and chi-square test. SPSS version 23 software (IBM Corporation NY, USA) was used when performing statistical analysis, with the level of significance being set at α = 0.05. In some cases, it was noticed that orbital fat tissue herniations and AEF were superposed. In all these cases, an enlargement in AEF was noted. AEFs with a diameter of least 3 mm and containing adipose tissue density were classified as orbital fat herniations. The upper limit of the normal anterior ethmoid foramen’s diameter was accepted as being 2.5 mm. In these instances, a positive Hounsfield Units (HU) density of artery-vein-nerve was noticed inside of them. In cases accepted as herniation, negative HU values of orbital adipose tissue were recorded in the AEFs. AEF enlargements that did not reach a diameter of 3 mm (<3 mm), which did not have fat tissue density, and were mostly observed as being symmetrical were not interpreted as herniations.

## 3. Results

A total of 688 and 512 people out of the 1200-person study group were male (57.3%) and female (42.7%), respectively. Their mean age was 38.74 and the interquartile range (IQR) of their ages was between 29 and 48 with a standard deviation of 12.511.

AEF in 2121 medial orbital walls could be visualized by CT (88.4%). If AEF was visualized, it was localized in Zones 2 or 3 in all cases, though it was generally close to the junction of Zones 2 and 3. In 2121 medial orbital walls where AEF could be visualized, the AEF rate in Zone 2 was 60.7% (1287), while the AEF rate in Zone 3 was 39.3% (834).

A total of 98 herniations were observed in 95 (7.9%) of the 2400 medial orbital walls of the 1200 patients. Herniation was unilateral in 92 patients, whereas it was bilateral in three patients.

**Figure 2:**
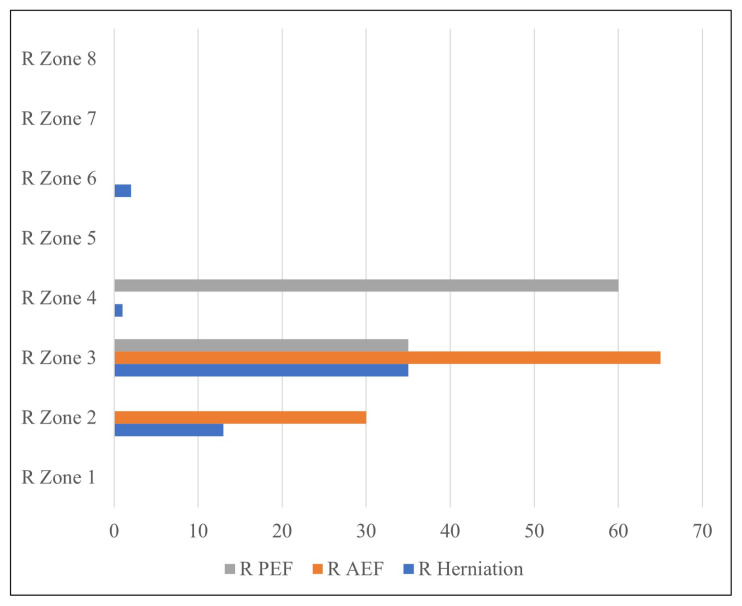
Distribution of herniations in the right medial orbital wall according to zones and AEF, PEF localizations.

**Figure 3:**
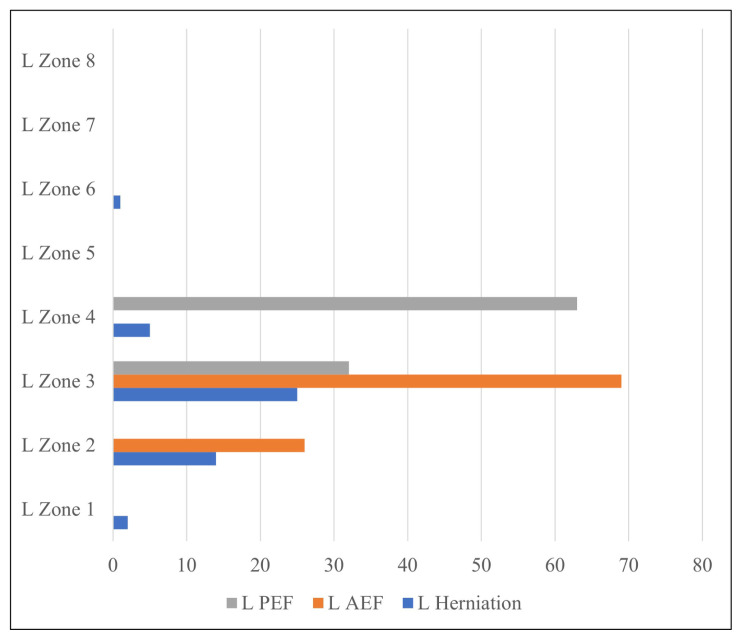
Distribution of herniations in the left medial orbital wall according to zones and AEF, PEF localizations.

Of the 51 herniations on the right medial orbital wall, 13 were in Zone 2, 35 were in Zone 3, 1 was in Zone 4, and 2 were in Zone 6. No herniations were detected in Zones 1, 5, 7, and 8 of the right medial orbital wall (Figure 2). Of the 47 herniations in the left medial orbital wall, 2 were recorded in Zone 1, 14 were recorded in Zone 2, 25 were recorded in Zone 3, 5 were recorded in Zone 4, and 1 was recorded in Zone 6. No herniation was recorded in Zones 5, 7, and 8 in the left medial orbital wall (Figure 3).

In the group with orbital fat tissue herniation, 30 of the right AEFs were localized in Zone 2 (31.6%), while 65 were localized in Zone 3 (68.4%). Moreover, while 35 of the right PEFs were localized in Zone 3 (36.8%), 63 of them were localized in Zone 4 (63.2%).

In the group with orbital fat tissue herniation, 26 of the left AEFs were localized in Zone 2 (27.4%) and 69 were localized in Zone 3 (72.6%). Furthermore, while 32 of the left PEFs were localized in Zone 3 (33.7%), 63 of them were localized in Zone 4 (66.3%).

Thirty-eight herniations on the right medial orbital wall were in the same zone as the right AEF and were likewise associated with the right AEF.

Thirty-two herniations in the left medial orbital wall were in the same zone as the left AEF and were similarly associated with the left AEF. Twelve herniations in the right medial orbital wall were in the same zone as the right PEF, and they were associated with the right PEF as well.

Eleven herniations in the left medial orbital wall were in the same zone as the left PEF and were, in addition, associated with the left PEF. The vertical size of the herniations was 2–17 mm (mean 6.8 mm), their transverse size was 2–12 mm (mean 5.7 mm), and their sagittal size was 3–30 mm (mean 10.8 mm).

**Figure 4:**
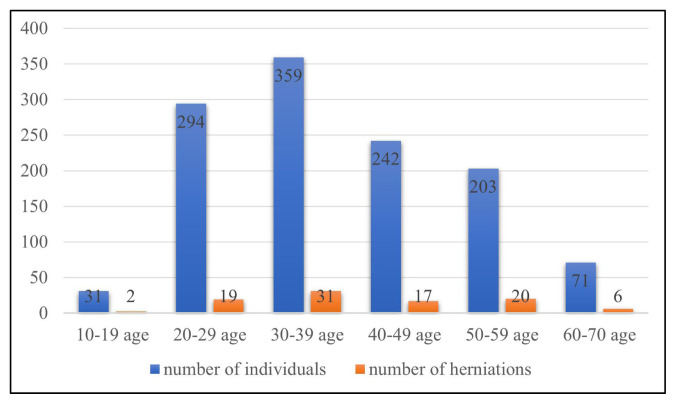
The number of patients with orbital fat tissue hernia in the medial orbital wall by age groups.

**Figure 5:**
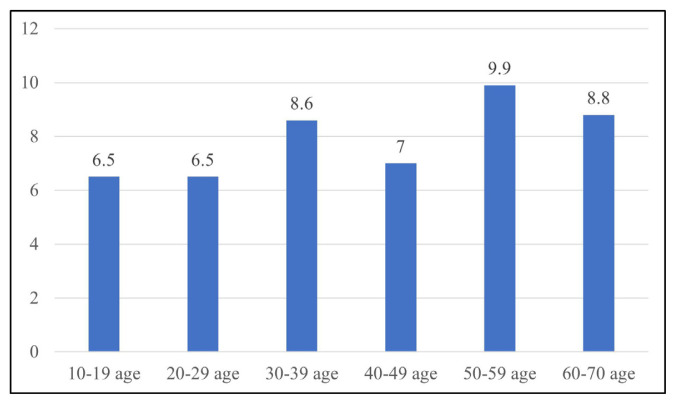
Orbital fat tissue herniation percentages by age groups (%).

While 96 of the herniations consisted only of fat tissue, the medial rectus muscle in one patient and the intraorbital vascular structure in another patient were accompanied by fat tissue. Out of the 1200 total patients studied in this research, the frequency of herniation was 6.5% in the 10–19 age group, 6.5% in the 20–29 age group, 8.6% in the 30–39 age group, 7% in the 40–49 age group, 9.9% in the 50–59 age group, and 8.8% in the 60–69 age group (Figure 4,5). The decadal distribution of patients with herniation is as follows: 2.1% between those 10–19 years old, 20% between those 20–29 years old, 32.6% between those 30–39 years old, 17.9% between those 40–49 years old, 21.1% between those 50–59 years old, and 6.3% between those 60–69 years old. In total, then, 95 out of the 1200 patients had instances of herniation.

Orbital fat tissue extending into the maxillary sinus through a defect in the orbital inferior wall was detected in one patient.

A statistically significant correlation was found between the anterior ethmoid foramen and orbital fat tissue herniations with the Spearman rank correlation coefficient test (r in range of 0.507–0.550, p < 0.001). No correlation was found between orbital fat tissue herniations and the posterior ethmoid foramen (r in range of −0.53 −0.268, p > 0.05) (Table). A statistically significant difference was noted between the localization zone of the AEF and the localization zones of orbital fat tissue herniations (Fisher’s exact test, p < 0.001).

## 4. Discussion

CT is the most appropriate examination for preoperative evaluation of sinonasal anatomy, especially when applied with thin-section protocols. Optimal imaging of anatomical variations and inflammatory changes reduces the risks that may occur during surgery [9].

Clinicians were able to gather detailed information about the anatomy and variations of the paranasal sinuses with CT, which leads to advances in surgical treatments, particularly functional endoscopic sinus surgery (FESS) [1].

Orbital fat tissue herniations in the medial orbital wall may be secondary to congenital or previous facial trauma and may sometimes mimic ethmoid sinusitis [12, 13].

Han et al. reported that the incidence of fat tissue herniation in the medial orbital wall was at the rate of 6.5% and that the rate increases with age. They reported the localization of orbital fat herniations in a decreasing manner, as in the superior, middle, and inferior parts of the LP [9]. Ohnishi and Yanagisawa also stated that bone dehiscences / orbital fat tissue protrusions can be seen at the medial and lateral ends of the AEF [14]. Kaya et al. emphasized the difference between dehiscence and orbital fat tissue herniations in the medial orbital wall [15]. Besides these limited studies, and although there is a relationship with orbital fat tissue herniations, especially with the AEF, it was unfortunately noticed that no authors paid attention to this relationship or their proximity in their studies.

The AEF and PEF are formations that show variations in terms of localization and number. Gottwald et al. visualized 95% of AEF in their CT study on plastinated cadaver specimens [16]. Our AEF visualization rate is 88.4%, which can be considered close to the rate of the aforementioned cadaver study. AEF visualization has been facilitated by the high spatial resolution obtained with thin slice thickness in developing CT devices and low pitch rates in multislice CTs.

**Figure 6:**
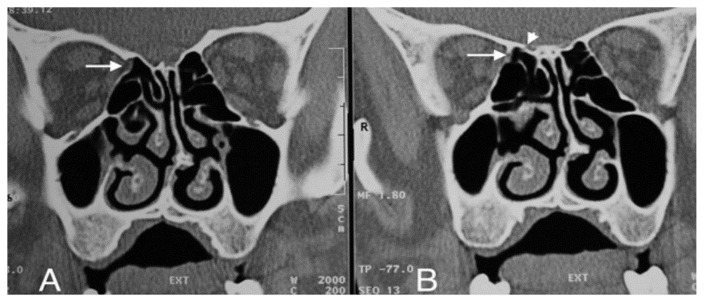
A- AEF-associated orbital fat tissue (white arrow) showing herniation from the right AEF level into the ethmoid sinus on the CT image. B- The orbital end of the ethmoidal canal (white arrow) and the cranial end (short white arrowhead) in the 3 mm posterior section.

**Figure 7:**
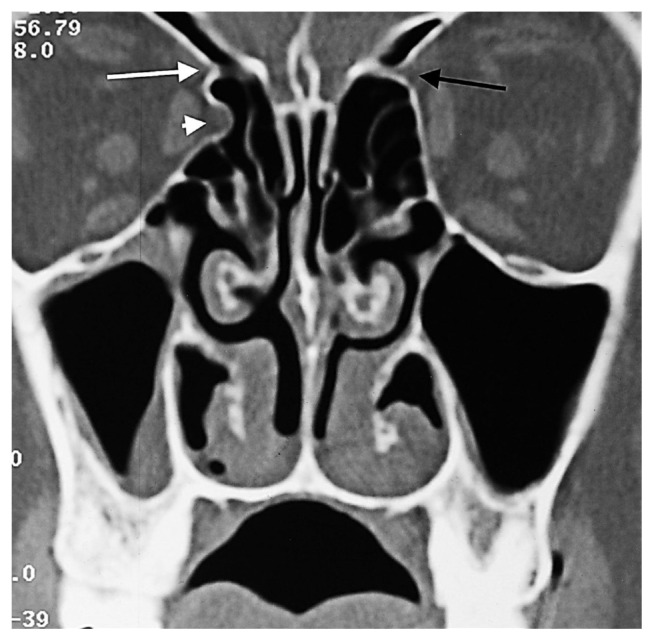
Herniated orbital fat tissue from Zone 3 to the anterior ethmoid sinus (short white arrowhead). This herniation has nothing to do with the ethmoidal foramen. Right AEF (long white arrow), left AEF (black arrow).

**Figure 8:**
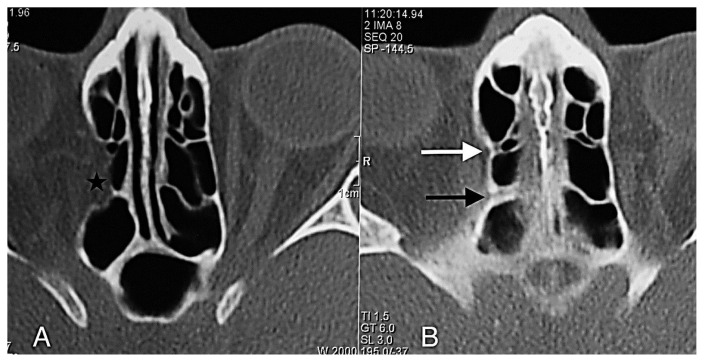
A- Orbital fat tissue (black asterisk) showing herniation from Zone 3 into the anterior and posterior ethmoid sinus; B- AEF (white arrow), PEF (black arrow) in the section passing 3 mm superior to section A.

**Figure 9:**
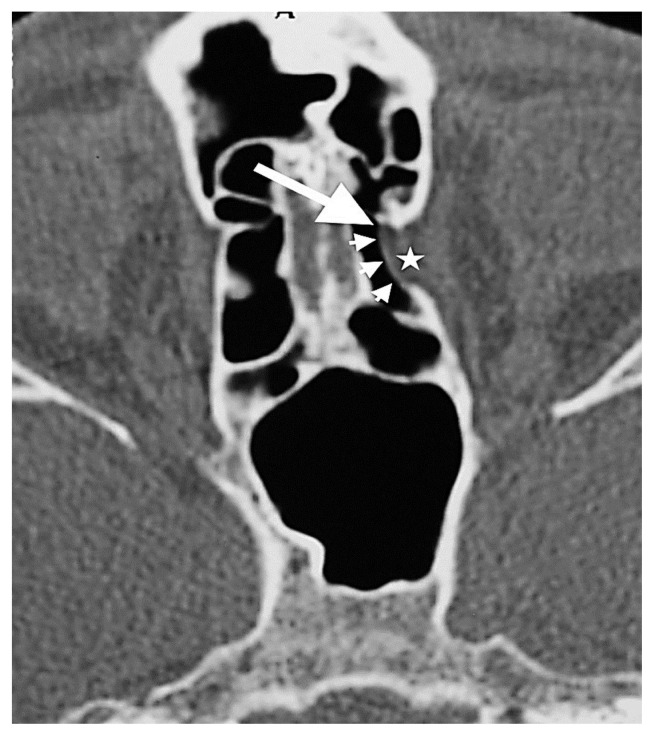
The orbital fat tissue (white asterisk), which is herniated from Zone 2 to the anterior ethmoid sinus, begins from the AEF (white long arrow) and extends to the posterior. There is a marked dehiscence in the medial orbital wall adjacent to the AEF (short white arrowheads).

**Figure 10:**
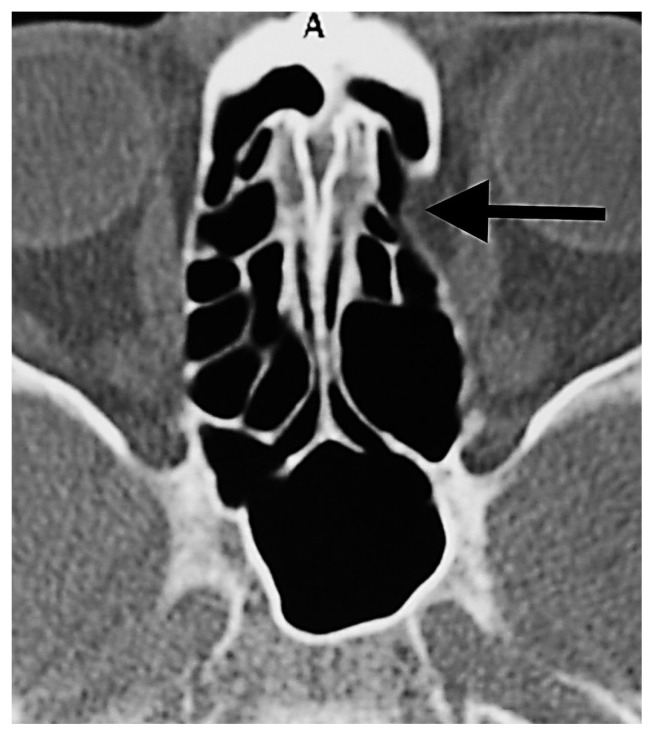
Orbital fat tissue associated with AEF showing herniation from Zone 2 to the anterior ethmoid sinus (black arrow).

The anterior ethmoidal canal is a bony canal that is thicker laterally and thinner medially (Figures 6A, 6B). The anterior ethmoidal canal is also called the orbitocranial canal [17] and is usually located in the second quarter where the axial plane is divided into four [18, 19, 20]. In our study, the most common location of AEF is Zone 2, which corresponds to the second quarter of the axial plane. In the group in which orbital fat tissue herniation was observed; however, AEFs were most frequently localized in Zone 3, followed by Zone 2 (Figures 7, 8A, 8B, 9, 10). This finding suggests that Zone 3 containing an AEF is the weakest area of the medial orbital wall and is the zone most susceptible to fat tissue herniations. It has been reported that the anterior ethmoidal canal can sometimes be buried in the ethmoid roof. Moreover, it is said that, if it is not noticed during FESS, it can be easily injured [14]. Indeed, this localization was thought to coincide with the peaks of Zones 2 and 3 in our study.

Moon et al. detected 11.4% dehiscence in the anterior ethmoidal canal itself in their cadaver study [2]. They attributed the high intra-canal dehiscence rate (40%) previously found by Kainz–Stammberger [21] to accepting small gaps belonging to anterior ethmoidal nerve and anterior ethmoidal veins passing through the anterior ethmoidal canal as dehiscence. These micro-dehiscences may be the cause behind the intra-AEF orbital fat tissue herniations that we found in our study and may cause an indirect increase in the diameter of AEFs.

**Figure 11:**
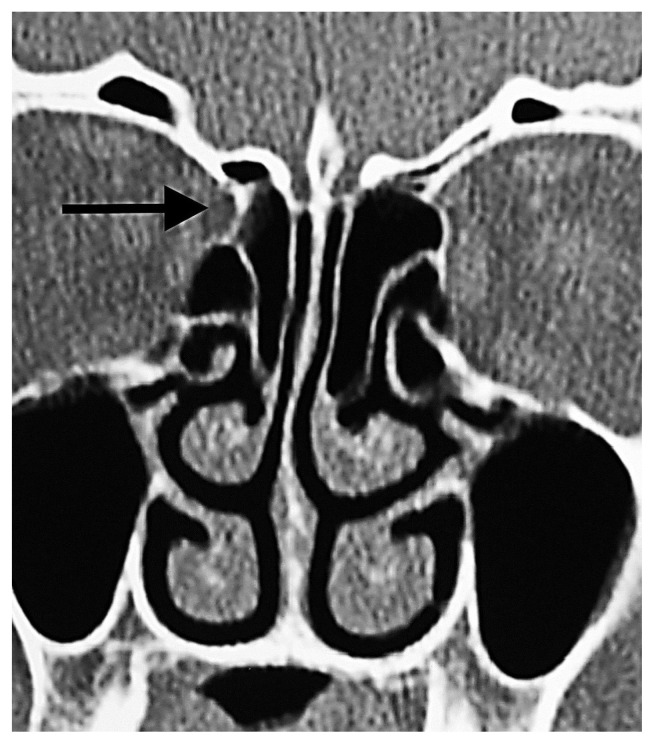
Orbital fat tissue showing herniation from Zone 3 to the anterior ethmoid sinus, disrupting the normal conical configuration of the AEF and causing enlargement in the AEF (black arrow).

**Figure 12:**
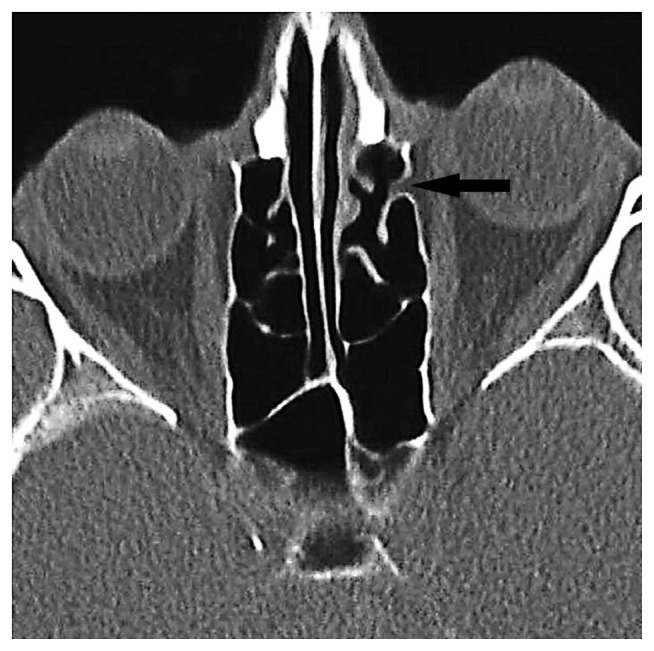
Orbital fat tissue herniated from Zone 1 to the anterior ethmoid sinus (black arrow). Herniation has nothing to do with the ethmoidal foramen.

**Figure 13:**
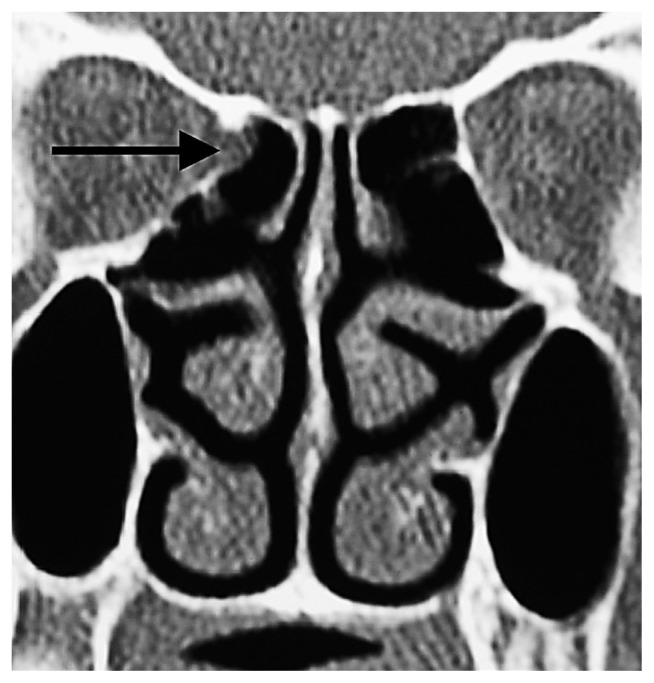
Orbital fat tissue associated with PEF, unrelated to AEF, showing herniation from Zone 4 to the posterior ethmoid sinus (black arrow).

**Figure 14:**
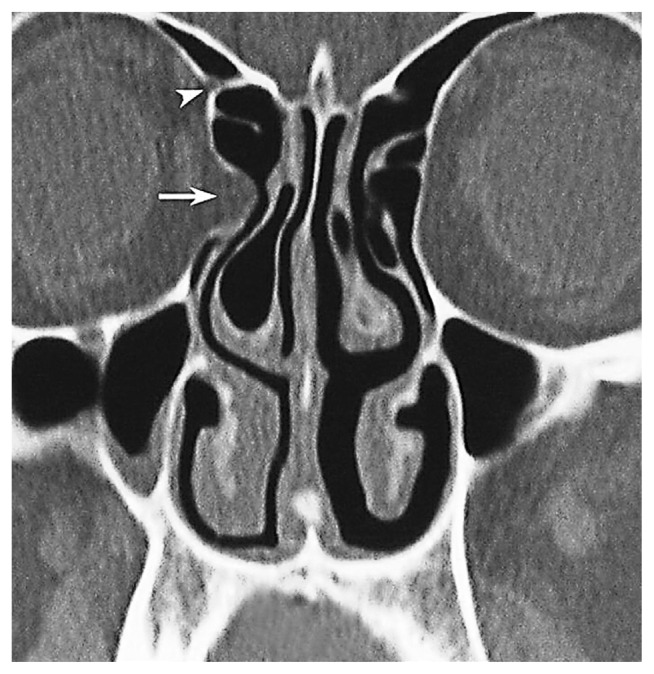
Orbital fat tissue showing herniation from zone 6 to the anterior ethmoid sinus (white arrow), AEF (white arrowhead). Herniation has nothing to do with the ethmoidal foramen.

The upper limit of a normal AEF diameter is expressed as 2.5 mm [22]. In our study, we evaluated AEFs with a canal diameter of at least 3 mm and in which we observed orbital fat tissue density as fat tissue herniation accompanied by dehiscence (Figure 11). It is clear, however, that cadaver studies will be more valuable for the definitive detection of orbital fat tissue herniations in enlarged ethmoidal foramina. This study’s novel finding is that fat tissue herniations in the medial orbital wall are localized in a statistically significant way within, or very close to, the AEF. More rarely, herniations with Zone 1, 4, and 6 localizations away from the AEF are also possible (Figures 12, 13, 14).

**Figure 15:**
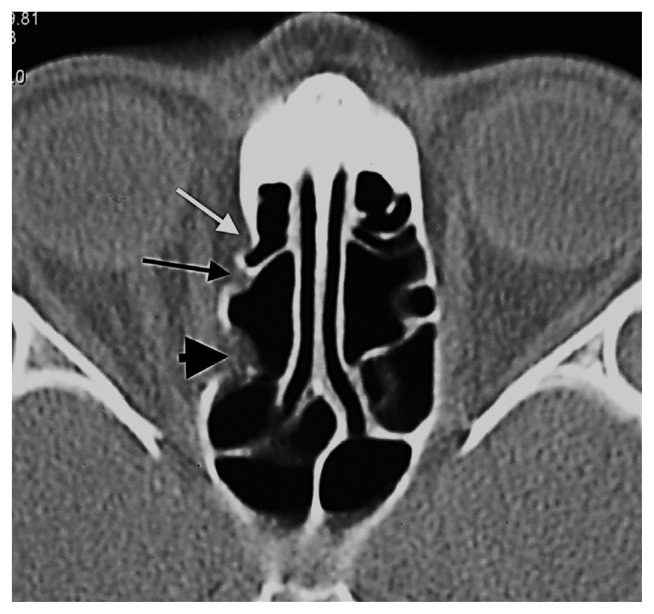
Orbital fat tissue showing herniation most prominently from Zone 3 and in three separate locations, to the anterior and posterior ethmoid sinuses (white arrow: anterior ethmoid herniation unrelated to AEF, long black arrow: anterior ethmoid herniation with AEF localization, black arrowhead: posterior ethmoid herniation unrelated to AEF, PEF).

**Figure 16:**
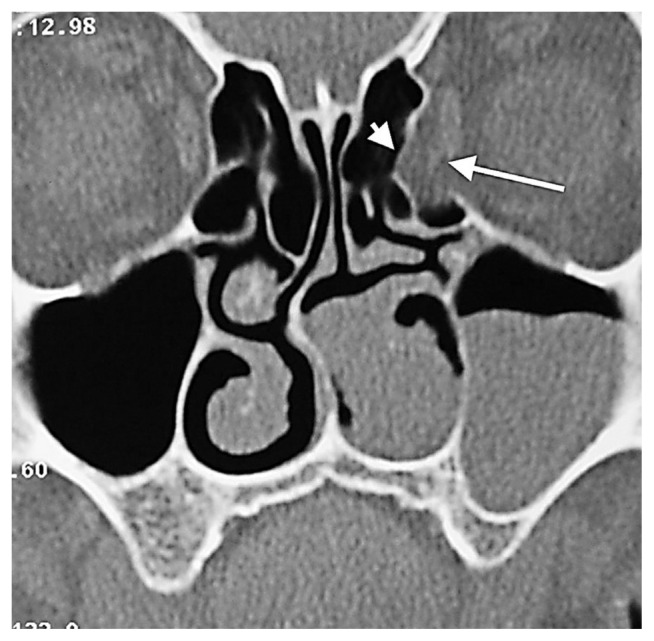
Orbital fat tissue (short white arrowhead) and orbital vein (long arrow) showing herniation from Zone 6 to the anterior ethmoid sinus which has nothing to do with the ethmoidal foramina.

In our study, it was shown that more than one orbital fat tissue herniation may occur on the same side (Figure 15). It is also the first study, which records the occurrence of an orbital vascular structure in the contents of a hernia (Figure 16). Orbital fat tissue herniations are observed as negative HU densities of 0 HU or close to 0 of the fat tissue observed in AEF or ethmoid cells. CT reports often do not contain key information needed to plan surgical procedures. Nevertheless, this information is necessary to prevent intracranial and intraorbital complications. Radiologists often do not pay attention to the information that surgeons may need before operations. Although surgeons expect to see AEFs 24 mm posterior to the anterior lacrimal crest of the frontoethmoidal suture, which is an important external landmark during FESS [23], orbital fat herniations in or near the AEF may cause difficulties in visualizing the AEF and locating the anterior ethmoidal artery in the AEF due to anatomical distortions. Therefore, it is recommended to carefully investigate orbital fat tissue herniations in radiological reporting before FESS.

The limitation of our study is that it is a retrospective cross-sectional study. Since our study is cross-sectional, it may not fully reflect the prevalence of orbital adipose tissue in the entire population.

As a result, in our study, we found that orbital fat tissue herniations mostly develop in and around the AEF. Knowing this may help surgeons better predict the anatomical view to be expected before FESS and, thus, minimize the risks of possible orbital complications (anterior ethmoidal artery injury, medial rectus muscle injury, and orbital cellulitis triggered by FESS).

## Figures and Tables

**Table:** The relationship between AEF, PEF localizations, and fat tissue herniations in the medial orbital wall (Spearman’s rank correlation coefficient).

Spearman’s Rho		R Hernia	L Hernia	R AEF	L AEF	R PEF	L PEF
**R Hernia**	**r**	1.000	.500	.507	.232	.268	.205
	** p**	.	.667	.000	.102	.058	.149
**L Hernia**	**r**		1.000	.074	.550	−.018	−.053
	** p**		.	.620	.000	.906	.724
**R AEF**	**r**			1.000	.599	.467	.378
	** p**			.	.000	.000	.000
**L AEF**	**r**				1.000	.216	.162
	** p**				.	.035	.117
**R PEF**	**r**					1.000	.933
	** p**					.	.000
**L PEF**	**r**						1.000
	** p**						.
